# Potential Treatments for COVID-19; a Narrative Literature Review

**Published:** 2020-03-21

**Authors:** Ali Rismanbaf

**Affiliations:** 1 Department of Clinical Pharmacy and Pharmacy Practice, School of Pharmacy and Pharmaceutical Sciences, Isfahan University of Medical Sciences, Isfahan, Iran.

**Keywords:** Coronavirus, Drug therapy, Clinical trial, Case reports, Review, COVID-19

## Abstract

SARS-CoV-2 is a newly emerging human infectious coronavirus that causes COVID-19, which has been recognized as a pandemic by the World Health Organization (WHO) on March 11^th^. There is still no vaccine or definitive treatment for this virus because its pathogenesis and proliferation pathways are still unknown. Therefore, in this article, new potential COVID-19 therapies are briefly reviewed.

## Introduction

SARS-CoV-2 (Severe Acute Respiratory Syndrome Coronavirus 2) is a newly emerging human infectious coronavirus, originated in Wuhan, China, and has been spreading rapidly in China and other countries since December 2019 (1). The World Health Organization (WHO) also declared a global emergency on January 31^st^ due to increasing concerns over its fast spread, and on March 11^th^ the disease was recognized as a pandemic. Since the bases for pathogenesis of this virus and its proliferation is unclear, there is still no vaccine or definitive treatment against it. Thus, medications used against SARS-CoV-2 are mainly based on their effectiveness on earlier strains of coronavirus, SARS-CoV and MERS-CoV. Therefore, the immediate introduction of potential COVID-19 treatments can be essential and salvaging. In this article, new potential COVID-19 therapies are briefly reviewed.

## Methodology

Articles were extracted, irrespective of time, using PubMed, Embase, and Google Scholar search engines, searching terms "COVID-19", "SARS-CoV-2", and "2019-nCOV" in titles, abstracts and keywords. Afterwards, clinical trials, clinical reports, case reports, and suggestions for potential medications against COVID-19 were briefly reviewed.

## Results

**Clinical reports**

Clinical reports on COVID-19 treatment mainly described empirical treatments and clinical experiences during its treatment. In 2020, Gao et al. studied the effect of chloroquine and hydroxychloroquine in treatment of COVID-19 in over 100 patients and 10 hospitals in Wuhan, Jingzhou, Guangzhou, Beijing, Shanghai, Chongqing, and Ningbo. The results of this study showed that chloroquine phosphate is effective in preventing the exacerbations of pneumonia, decreasing lung involvements in imaging findings, promoting a virus-negative conversion and shortening the disease course. In addition, there were no serious adverse effects observed at therapeutic doses (2).

**Table 1:** Potential drugs for COVID-19

**Study**	**Method**	**Medicine**	**Mechanism of Action**
Wang et al. (2020) (12)	In vitro study	Chloroquine Remdesivir	Reducing viral copy numbers in the cell supernatant and viral infection
Zhang et al. (2020) (13)	In vitro study	Teicoplanin	Preventing the entrance of SARS-CoV-2-Spike-pseudoviruses into the cytoplasm
Xu et al. (2020) (14)	Virtual screening	Nelfinavir	Binding to SARS-CoV-2 M^pro^
Liu et al. (2020) (15)	Virtual screening	Colistin Valrubicin Icatibant Bepotastine Epirubicin Epoprostenol Vapreotide Aprepitant Caspofungin perphenazine	Binding to SARS-CoV-2 M^pro^
Shang et al. (2020) (16)	Virtual screening	Rupintrivir Lopinavir Remdesivir	Binding to SARS-CoV-2 M^pro^
Jin et al. (2020) (17)	Virtual screening	Ebselen	Binding to SARS-CoV-2 M^pro^
Sekhar et al. (2020) (18)	Virtual screening	Beclabuvir Saquinavir	Binding to SARS-CoV-2 M^pro^
Contini et al. (2020) (19)	Virtual screening	(Angiotensin II human acetate) GHRP-2 Indinavir Cobicistat Caspofungin acetate Lopinavir Atazanavir	Binding to SARS-CoV-2 M^pro^: Angiotensin II human acetate, GHRP-2, Indinavir, and Cobicistat Binding to SARS-CoV-2 3C-like proteinase (3CL^pro^): Angiotensin II human acetate, GHRP-2, Indinavir, Caspofungin acetate, Lopinavir, and Atazanavir
Wang et al. (2020) (20)	Virtual screening	Carfilzomib Eravacycline Valrubicin Lopinavir Elbasvir Streptomycin	Binding to SARS-CoV-2 protease
Wang et al. (2020) (21)	Virtual screening	Thymopentin Carfilzomib Saquinavir	Binding to SARS-CoV-2 3C-like proteinase (3CL^pro^)
Chen et al. (2020) (22)	Virtual screening	Ledipasvir velpatasvir	Binding to SARS-CoV-2 3C-like proteinase (3CL^pro^)
Beck et al. (2020) (23)	Molecule Transformer-Drug Target Interaction (MT-DTI)	Atazanavir Efavirenz Ritonavir Dolutegravir	Binding to SARS-CoV-2 3C-like proteinase (3CL^pro^)
Elfiky et al. (2020) (24)	Virtual screening	Mycophenolic acid Grazoprevir Telaprevir Boceprevir	Binding to SARS-CoV-2 papain-like protease (PL^pro^)
Arya et al. (2020) (25)	Virtual screening	Formoterol Chloroquine	Binding to SARS-CoV-2 papain-like protease (PL^pro^)
Smith et al. (2020) (26)	Virtual screening	Eriodictyol Isoniazid pyruvate Nitrofurantoin Cepharanthine Ergoloid Hypericin	Binding potency to Viral S-protein at its host receptor region or to the S protein-human ACE2 interface
Li et al. (2020) (27)	Connectivity map (Cmap)	Ikarugamycin molsidomine	Effective on the genes co-expressed with ACE2
Richardson et al. (2020) (28)	Using BenevolentAI	Baricitinib	Binding to AP2-associated protein kinase 1 (AAK1)
Nowak et al. (2020) (29)	Brief review	Lithium	Probably by reducing apoptosis and inhibition of glycogen synthase kinase 3 beta (GSK-3β)
Sun et al. (2020) (30)	Brief review	Angiotensin converting enzyme inhibitors and Angiotensin1 receptor inhibitors	Rebalancing Renin-Angiotensin-Aldosterone System (RAAS) (might reduce the pulmonary inflammatory response and mortality)

Also, according to Jian-ya et al., treatment of 51 COVID-19 patients with traditional Chinese medicine, interferon, Lopinavir, Ritonavir and short-term (3 to 5 days) corticosteroids was successful and resulted in recovery and discharge of 50 patients (3). Qin et al. also reported that administration of moxifloxacin, lopinavir, and interferon to non-ICU patients and the addition of methylprednisolone to the above treatment for ICU patients resulted in 26 patients being discharged from intensive care unit (ICU) and 16 patients being discharged from hospital (4). Also, Zhou et al. reported that short-term moderate-dose corticosteroid (160 mg/day) plus immunoglobulin (20 g/day) significantly reduced lung injury, normalized lymphocyte counts, body temperature, C-reactive protein levels, and oxygenation index in 10 COVID-19 patients (5). On the other hand, while studying 416 COVID-19 patients, Shang et al. reported that corticosteroid therapy and gamma globulin administration increased mortality and appeared to be useful only in patients with lower lymphocyte counts (6). According to the mentioned clinical reports, the administration of corticosteroids for COVID-19 patients is still questionable.

**Case reports**

So far, there are three published case reports on the successful treatment of patients with COVID-19. In the first report Lim et al. described a 54-year-old man with COVID-19 who was treated with Lopinavir/Ritonavir from day 10 of illness, 2 tablets (Lopinavir 200mg / Ritonavir 50mg) every 12 hours. Since first day of administration, β-coronavirus viral load started to decrease, and little or no detectable coronavirus titers have been observed since then (7).

In another case report, Zhang et al. described a couple who were both 38 years old and were suffering from COVID-19. Their treatment included Methylprednisolone 40 mg daily intravenous (IV) injections for one and five days for the male and the female patient respectively, human gamma globulin 10g IV qd for five and seven successive days for the male and the female patient, respectively, and then the dose was changed to 5g for both of them, in addition to Moxifloxacin, Oseltamivir, Arbidol hydrochloride, and Tanreqing (Chinese patent medicine). After 11 days, the female patient and after 14 days the male patient recovered with regards to inflammatory factors and were discharged from the hospital (8).

In the third case report, Chen et al. reported a 45-year-old woman with COVID-19 and stated that after treatment with Thalidomide (100 mg orally once a day) and Methylprednisolone (40 mg intravenously bid for 3 days then reduced to once a day for 5 days) the overall patient status was improved, oxygen index was increased, symptoms of nausea and vomiting were alleviated, and cytokine levels were decreased (9).

**Potential drugs**

Several articles have suggested medicines, potentially effective for the treatment of COVID-19 (Table 1). Most of these suggestions are based on in vitro studies, virtual screenings and records of their effects on SARS and MERS.

In addition to these medications, Tocilizumab has recently been suggested as a COVID-19 treatment. Studies have shown that IL-6 levels significantly correlated with the severity of COVID-19, C-reactive protein (CRP), lactate dehydrogenase (LDH), and D-dimer levels and T cell counts, and it has been suggested that Tocilizumab, with its inhibitory effect on IL-6, may be effective in treatment of COVID-19 (10, 11). However, no clinical study has demonstrated the effects of Tocilizumab on COVID-19 and further studies are indeed required.

## Conclusion

Apparently, in addition to the drugs currently prescribed to treat COVID-19, Arbidol hydrochloride, interferon, and Thalidomide plus Methylprednisolone can also be used due to their effects reported in clinical studies. However, more studies are needed to confirm the use of corticosteroids, as there are conflicting reports regarding their efficacy. Also, potential drugs listed in Table 1, such as Remdesivir, Atazanavir, Saquinavir, and Formoterol, and Tocilizumab can be introduced as treatments for COVID-19 if they prove to be effective in animal and clinical studies.
